# Effect of food intake on left ventricular wall stress

**DOI:** 10.1186/1476-7120-12-2

**Published:** 2014-01-28

**Authors:** Ylva Gårdinger, Joanna Hlebowicz, Ola Björgell, Magnus Dencker

**Affiliations:** 1 Department of Clinical Sciences, Unit of Clinical Physiology and Nuclear Medicine, Skåne University Hospital, Lund University, Malmö, Sweden; 2 Department of Clinical Sciences, Unit of Radiology, Skåne University Hospital, Lund University, Malmö, Sweden; 3 Department of Clinical Sciences, Division of Medicine, Skåne University Hospital, Lund University, Malmö, Sweden

**Keywords:** Food intake, Echocardiography, Wall stress

## Abstract

**Objective:**

Left ventricular wall stress has been investigated in a variety of populations, but the effect of food intake has not been evaluated. We assessed whether left ventricular wall stress is affected by food intake in healthy subjects.

**Methods:**

Twenty-three healthy subjects aged 25.6 ± 4.5 years were investigated. Meridional end-systolic wall stress (ESS) and circumferential end-systolic wall stress (cESS) were measured before, 30 minutes after, and 110 minutes after a standardised meal.

**Results:**

Both ESS and cESS decreased significantly (P < 0.001) from fasting values 30 minutes after the meal, and had not returned to baseline after 110 minutes. ESS decreased from 65 ± 16 kdynes/cm^2^ (fasting) to 44 ± 12 kdynes/cm^2^ 30 minutes after, and to 58 ± 13 kdynes/cm^2^ 110 minutes after eating. cESS decreased from 98 ± 24 kdynes/cm^2^ to 67 ± 18 kdynes/cm^2^ 30 minutes after, and to 87 ± 19 kdynes/cm^2^ 110 minutes after the meal.

**Conclusion:**

This study shows that left ventricular wall stress is affected by food intake in healthy subjects.

## Introduction

End-systolic wall stress has been used as a measurement of myocardial afterload, the counter force limiting left ventricular ejection. To put it simply, circumferential fiber shortening is limited by circumferential end-systolic wall stress (cESS), whereas longitudinal fiber shortening is limited by meridional end-systolic wall stress (ESS) [1,2]. Left ventricular wall stress has been examined in a number of populations, for example in patients with hypertensive heart disease [3,4], in patients with type 1 diabetes mellitus [5] and patients under treatment with beta-1 blockers [6]. Wall stress has also been shown to have prognostic value [4]. Digestion of food is known to significantly alter hemodynamics [7-10] and may therefore affect wall stress, as loading conditions are altered. The purpose of the present study is to evaluate the hypothesis that food intake, in healthy volunteers, may have an effect on meridional and circumferential wall stress, as this has not previously been investigated.

## Methods

### Study population

Study subjects were comprised of 23 healthy volunteers (11 male and 12 female aged 25.6 ± 4.5 years). No subjects had symptoms or history of cardiovascular disease or any other chronic diseases. None of the subjects were taking cardiovascular medication. Other exclusion criteria were inappropriate acoustic windows and non-sinus rhythm.

### Procedures

The examinations were performed in the morning after fasting overnight. Height and body mass were measured, and body mass index was calculated. The following formula was used to calculate body surface area (BSA): (height in cm) 0.725 × (body mass in kg) 0.452 × 0.00718 [11]. Blood pressure (BP) and resting heart rate were measured in the supine position after 15 minutes of rest. BP was measured by a conventional (mechanical) sphygmomanometer with aneroid manometer and stethoscope. Systolic pressure (SBP) (first phase) was identified with the first of the continuous Korotkoff sounds. Diastolic pressure was identified at the moment the Korotkoff sounds disappear (fifth phase). A baseline echocardiographic exam was performed, after which the subjects ingested a standardised meal consisting of 300 g rice pudding (AXA Goda Gröten Risgrynsgröt; Lantmännen AXA, Järna Sweden). A second echocardiographic exam was performed 30 minutes after, and a third exam 110 minutes after the meal. The subjects reassumed a supine position between the echocardiographic examinations. The study was approved by the Central Ethical Review Board Lund, Sweden.

### Echocardiography

After an initial screening examination to rule out cardiac dysfunction, a transthoracic echocardiographic examination was performed before, 30 minutes, and 110 minutes after the meal, with Sonos 5500 (Philips, Andover, MA, USA) in left lateral position, after 15 minutes rest. A single observer performed all echocardiographic measurements three times on separate cardiac cycles, and the mean value was used in analyses. Repeated measurements in all subjects were performed to assess intra-observer reproducibility. In 2D guided M-mode echocardiography from parasternal long axis view, the left ventricular internal dimension in diastole (LVIDd) and systole (LVIDs) and the posterior wall thickness in systole (PWTs) were measured with the ultrasound beam at or just below the tips of mitral valve leaflets. The measurements were made according to the American Society of Echocardiography guidelines [12].

Meridional end-systolic stress was calculated using the following formula, which is invasively validated, [3,13] where SBP is systolic blood pressure.

$$\mathrm{ESS}=\frac{0.334\times \mathrm{SBP}\times \mathrm{LVIDs}}{\mathrm{PWTs}\times \left\{1+\left(\mathrm{PWTs}/\mathrm{LVIDs}\right)\right\}}$$

Circumferential end-systolic stress was also estimated from M-mode tracings as previously described [3,14].

$$\mathrm{cESS}=\frac{\mathrm{SBP}\times {\left(\mathrm{LVIDs}/2\right)}^{2}\times \left\{1+\frac{{\left(\mathrm{LVIDs}/2+\mathrm{PWTs}\right)}^{2}}{{\left(\mathrm{LVIDs}/2+\mathrm{PWTs}/2\right)}^{2}}\right\}}{{\left(\mathrm{LVIDs}/2+\mathrm{PWTs}\right)}^{2}-{\left(\mathrm{LVIDs}/2\right)}^{2}}$$

### Statistical analysis

Data are presented as mean ± standard deviation (SD). Statistical analyses were performed using Statistica 7.1 (StatSoft Inc, Tulsa, OK, USA). Comparisons between fasting values for ESS and cESS, versus values 30 and 110 minutes after the ingestion of food, were analyzed for significance with Wilcoxon matched pairs test. Statistical significance was set at a level of P < 0.05.

## Results

All subjects had complete measurements, and no subjects were found to have any cardiac dysfunction. Table 1 displays descriptive statistics of the study population and cardiac dimensions.

**Table 1:** Subjects’ anthropometric characteristics and cardiac dimensions (n = 23)

**Variable**	
Sex (male/female)	11/12
Body mass (kg)	68 ± 11
Height (cm)	177 ± 8
BMI (kg/m^2^)	21.7 ± 2.2
BSA (m^2^)	1.8 ± 0.2
LVIDd (mm/m^2^)	26.7 ± 1.9
LVIDs (mm/m^2^)	17.7 ± 1.9
IVSd (mm/m^2^)	5.1 ± 0.4
PWTd (mm/m^2^)	5.0 ± 0.4
PWTs (mm/m^2^)	6.9 ± 0.9
LA (mm/m^2^)	18.2 ± 1.9
LVM (g/m^2^)	87.6 ± 18.1

Values are mean ± SD.

*Abbreviations*: Indexed for BSA: left ventricular internal dimension in diastole (LVIDd), left ventricular internal dimension in systole (LVIDs), interventricular septum thickness in diastole (IVSd), posterior wall thickness in diastole (PWTd), posterior wall thickness in systole (PWTs), left atrial end-systolic diameter (LA), and left ventricular mass (LVM). Body surface area (BSA).

**Figure 1:**
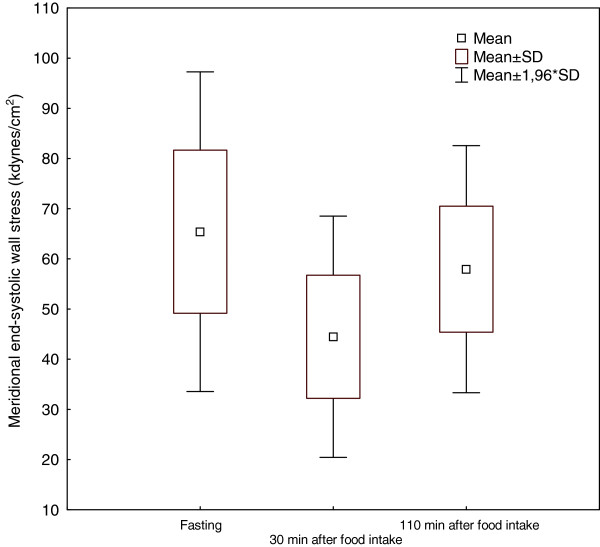
Meridional end-systolic wall stress before, 30 minutes after, and 110 minutes after the meal.

**Figure 2:**
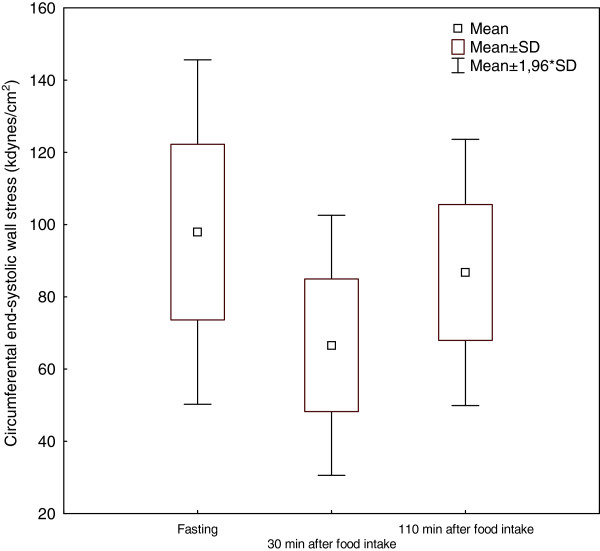
Circumferential end-systolic wall stress before, 30 minutes after, and 110 minutes after the meal.

The mean value for PWTs increased 30 minutes after food intake, and had almost returned to the fasting value 110 minutes after eating. The mean value for LVIDs decreased 30 minutes after the meal, and was still somewhat below the fasting value after 110 minutes. Systolic BP was not significantly altered after food intake, whereas diastolic BP decreased from 66 ± 7 mmHg fasting to 58 ± 7 mmHg 30 minutes after the meal, and 63 ± 6 mmHg 110 minutes after food intake. Both ESS and cESS decreased significantly (P < 0.001) from fasting values 30 minutes after the meal, and had not returned to baseline values 110 minutes after food intake (Figures 1 and 2). Both ESS and cESS were found to decrease 32% 30 minutes after the meal, and 110 minutes after food intake these values were still decreased by 11%, compared to fasting values (Table 2). Intra-observer variability was 10% for both ESS and cESS.

**Table 2:** Hemodynamics, blood pressure and heart rate parameters before, 30 minutes after, and 110 minutes after a standardized meal (n = 23)

**Variable**	**Fasting**	**30 minutes after food intake**	**110 minutes after food intake**
ESS (kdynes/cm^2^)	65 ± 16	44 ± 12***	58 ± 13*
cESS (kdynes/cm^2^)	98 ± 24	67 ± 18***	87 ± 19**
LVIDs (mm)	32 ± 3	28 ± 4***	31 ± 3*
PTWs (mm)	12.6 ± 2.0	14.7 ± 2.1***	13.2 ± 1.7
IVSs (mm)	12.9 ± 1.8	15.3 ± 2.0***	13.7 ± 1.9
Heart rate (bpm)	60 ± 8	64 ± 10**	60 ± 10
Systolic BP (mmHg)	103 ± 9	102 ± 10	102 ± 9
Diastolic BP (mmHg)	66 ± 7	58 ± 7***	63 ± 6*

Values are mean ± SD.

*Abbreviations*: Blood pressure (BP), meridional end-systolic wall stress (ESS), circumferential end-systolic wall stress (cESS), left ventricular inner diameter in systole (LVIDs), posterior wall thickness in systole (PWTs), and septal wall thickness in systole (IVSs). Please observe that values for cardiac size are reported in absolute values.

*Indicates significant difference (P < 0.05), compared to fasting values.

**Indicates significant difference (P < 0.01), compared to fasting values.

***Indicates significant difference (P < 0.001), compared to fasting values.

## Discussion

This study shows that left ventricular wall stress is affected by food intake, and even 110 minutes after food intake, wall stress had not returned to fasting values*.* Since hemodynamics is known to change postprandially, it is not unreasonable that wall contractility would also be altered accordingly.

In our study population, systolic BP was not significantly altered after a meal, whereas both posterior wall thickness (PWTs) and ventricular inner diameter (LVIDs) changed, resulting in significant changes in both meridional- and circumferential end-systolic wall stress. Thus, the findings are not driven by blood pressure but by myocardial contractility. The mechanisms behind the findings in the present investigation are not known. Several kinds of postprandial cardiovascular changes have, however, been reported in the literature. Postprandial cardiac output increase has been suggested to result from increases in blood flow in the superior mesenteric artery, the heart rate and stroke volume [9]. Physiological changes in the levels of glucose, insulin, GLP-1 and ghrelin may also influence the activity of the heart [15]. Moreover, it is known that insulin has positive chronotropic and inotropic effects on the heart [16], and the hormone glucagon-like peptide 1 has been shown to increase left ventricular function [17,18]. The hormone ghrelin has been shown to increase cardiac output and stroke volume [19-22].

As end-systolic wall stress has been used as a measurement of myocardial afterload, it is not surprising that changes are seen after food intake. The effect of food intake on left ventricular wall stress has not been investigated previously. The effect of alterations in loading condition on left ventricular wall stress has, however, been investigated by means of fluid loading and application of sublingual glyceryl trinitrate in a population of patients that were undergoing routine coronary angiography [23]. Fluid loading increased wall stress, and application of glyceryl trinitrate decreased wall stress. This corresponds well to the hypothesis of postprandial hemodynamic changes affecting wall stress.

Left ventricular wall stress and mid**-**wall mechanics have been used to investigate different populations, including patients with ventricular hypertrophy [24], hypertensive heart disease [3,4], diabetes [5], and patients undergoing treatment with beta blockers [6]. There have also been studies investigating age-related changes in wall stress [25,26]. The time frames between food intake and echocardiographic examinations have, however, not been specified in these studies, suggesting that this was not controlled. The change in left ventricular wall stress seen after food intake are about the same magnitude as the differences seen between different populations (for example, athletes or hypertensive subjects versus controls) in some investigations [3,24]. While it is difficult to control patients’ food intake in clinical echocardiography, one should be aware of the effect that food intake has on these echocardiographic parameters*,* affecting in this case both ESS and cESS. The influence of food consumption should be considered in studies, especially when a small sample size is involved.

This investigation was limited by our inability to perform the echocardiographic exams blinded to the state of food intake, because a single observer performed all exams. In an attempt to avoid bias, all exams were stored digitally and the measurements were performed later in random order. The change in left ventricular wall stress had not yet returned to baseline values 110 minutes after food intake, and in hindsight we would have chosen a longer time period after eating. Moreover, we did not include a control group who did not receive the prepared meal after overnight fasting. The present study showed the effect of food intake only in young, healthy subjects. Additional studies are warranted in older healthy subjects and in patients with various health conditions to determine whether the findings in the present investigation are reproducible in such populations. Further studies are also needed to evaluate the time frame necessary for measurements to return to baseline values.

## Conclusion

This study shows that left ventricular wall stress is affected by food intake in healthy subjects.

## Competing interests

The authors declare that they have no competing interests.

## Authors’ contributions

The authors’ contributions were as follows: JH, OB and MD designed the study. JH was responsible for recruiting the subjects. MD performed the echocardiographic examinations. YG and MD carried out the statistical calculations. YG wrote the first draft of the manuscript, all authors made critical revisions of the manuscript. All authors read and approved the final manuscript.
